# High Sensitivity and High Stability QCM Humidity Sensors Based on Polydopamine Coated Cellulose Nanocrystals/Graphene Oxide Nanocomposite

**DOI:** 10.3390/nano10112210

**Published:** 2020-11-05

**Authors:** Yao Yao, Xianhe Huang, Qiao Chen, Zhen Zhang, Weiwei Ling

**Affiliations:** 1 School of Automation Engineering, University of Electronic Science and Technology of China, Chengdu 611731, China; xianhehuang@uestc.edu.cn (X.H.); qiaochen@std.uestc.edu.cn (Q.C.); 2 SCNU-TUE Joint Lab of Device Integrated Responsive Materials (DIRM), South China Academy of Advanced Optoelectronics, South China Normal University, Guangzhou 510006, China; 3 State Key Laboratory of Electronic Thin Films and Integrated Devices, University of Electronic Science and Technology of China, Chengdu 610054, China; lingweiwei@cuit.edu.cn

**Keywords:** humidity sensor, quartz crystal microbalance, polydopamine, cellulose nanocrystal, graphene oxide, high stability

## Abstract

In this paper, a high sensitivity and high stability quartz crystal microbalance (QCM) humidity sensor using polydopamine (PDA) coated cellulose nanocrystal (CNC)/graphene oxide (GO) (PDA@CNC/GO) nanocomposite as sensitive material is demonstrated. The PDA@CNC was prepared by the self-polymerization action on the surface of CNC, and it acted as filler material to form functional nanocomposite with GO. The material characteristics of PDA@CNC, CNC/GO and PDA@CNC/GO were analyzed by transmission electron microscope (TEM) and Fourier transform infrared spectroscopy (FTIR), respectively. The experimental results show that the introduction of PDA@CNC into GO film not only effectively enhanced the sensitivity of GO-based nanocomposite-coated QCM sensor but also significantly maintained high stability in the entire humidity range. The PDA@CNC/GO30-coated QCM humidity sensor exhibited a superior response sensitivity up to 54.66 Hz/% relative humidity (RH), while the change rate of dynamic resistance of the sensor in the humidity range of 11.3–97.3% RH is only 14% that is much smaller than that of CNC/GO-coated QCM. Besides, the effect of the PDA@CNC content on the sensitivity and stability of GO-based nanocomposite-coated QCM humidity was also studied. Moreover, other performances of PDA@CNC/GO-coated QCM humidity sensor, including humidity hysteresis, fast response and recovery and long-term stability, were systematically investigated. This work suggests that PDA@CNC/GO nanocomposite is a promising candidate material for realizing high sensitivity and high stability QCM humidity sensor in the entire humidity detection range.

## 1. Introduction

The precious measurement of humidity level plays a critical and increasing role in modern industry and daily life, such as industrial process control, electrostatic protection, SF_6_ gas leakage monitoring, grain storage, weather forecast, etc. [1,2] To meet this growing demand of the development of Internet of Things (IoT) [3,4], many types of transducers including resistance [5], capacitive [6], mechanical [7,8] and microwave [9] have been adopted to develop humidity sensors. Among them, quartz crystal microbalance (QCM), which is classified as mechanical type, is a powerful sensing platform with large sensitivity, high frequency stability, low cost and real-time monitoring [10,11,12]. Benefiting from its superior ability to detect nanogram mass variations, QCM can easily achieve high humidity response sensitivity by functionalizing the electrode with humidity sensitive materials. The adsorption or desorption of water molecules on sensitive material caused by humidity variations will change the surface mass (Δ*m*) of QCM electrode, thus changing the resonance frequency (Δ*f*) of QCM according to Sauerbrey’s relationship as shown in Equation (1) [13]. Here, *f*_0_ is the fundamental resonance frequency and *A* is the electrode area of QCM.

(1) $$\Delta f=-2.26\times 10^{-6}f_{0}^{2}\frac{\Delta m}{A}$$

As is known, the sensitivity is a key parameter to evaluate the performance of QCM humidity sensors. In the past two or three decades, many researchers have made a lot of efforts to improve the sensitivity of QCM humidity sensors, especially in the development of humidity sensitive materials. Various kinds of materials including ceramics [14], organic polymers [15] and carbon [16,17] have been reported to fabricate QCM humidity sensors so far. To further improve the sensitivity of QCM humidity sensors, there are two generally recognized approaches to modify sensitive materials. One is to treat the structure of sensitive materials to achieve the nanoscale, which can greatly improve the molecular adsorption area of the sensitive materials [18]. The other is to modify the sensitive material to increase the number of molecular adsorption active sites [19,20]. Notably, both approaches can achieve the improvement of sensor sensitivity through increasing water molecular adsorption capacity of sensitive materials. However, several researchers have found that some QCM humidity sensors have abnormal frequency responses that deviate from Sauerbrey’s relationship when many water molecules are adsorbed on the sensitive material [18,21,22]. For example, Wang experimentally found that PEI-coated QCM humidity sensors showed abnormal frequency responses (e.g., positive frequency shifts) in the high humidity range [18]. Erol observed that ZnO nanowire-coated QCM exhibited a positive frequency shift as the increase in humidity [21]. Üzar also found a similar experimental phenomenon that ZnS nanowires loaded on QCM presented a positive frequency shift at high humidity [22]. The above results suggest that the frequency stability of QCM humidity sensors should be especially concerned when enhance the QCM sensor’s sensitivity by increasing water molecular adsorption capacity of sensitive materials. Further study shows that the abnormal frequency response of QCM sensor is due to the viscoelastic properties change of the sensitive materials after water absorption [22]. Therefore, the physical and chemical properties of humidity sensitive materials are crucial to the performance of QCM humidity sensor, because they not only determine the sensitivity but also influence the frequency stability of the sensor.

In our previous works, we reported that graphene oxide (GO), an important derivative of two-dimensional graphene, was a good candidate material for realizing high performance QCM humidity sensors [23]. The structural model of GO is composed of a layer of hydrophobic carbon six-membered rings and a large number of hydrophilic groups (such as hydroxyl, epoxy and carboxyl) bonded to carbon layer [24]. The water adsorption of GO mainly occurs at the hydrophilic groups. Since the main structure of GO is hydrophobic carbon six-membered rings layer with high mechanical stiffness, GO-coated QCM humidity sensor showed high frequency stability [25]. However, it should also be noted that its sensitivity is not enough to meet the demand of highly sensitive humidity detection, especially at low humidity. To solve this problem, researchers have carried out a series of works to improve the sensitivity of GO-based QCM humidity sensor [26,27,28,29,30,31]. By the modification of GO with organic polymers [26,27], inorganic metal oxide particles [28,29] and carbonaceous nanoparticles [30,31], the obtained functional GO-based QCM humidity sensors possessed satisfactory humidity response sensitivity. However, these sensors still show the drawback of sharply decreasing frequency stability in the high humidity range. This is possibly because the above GO-based composite does not form strong adhesive force between GO and filler material; the swelling of GO after many water molecules are adsorbed will increase the dissipation of the QCM. Therefore, it remains a huge challenge to improve the sensitivity of GO-based QCM humidity sensors while ensuring high stability. Cellulose nanocrystal (CNC), as a biodegradable and nontoxic nanomaterial, has been proved to be an excellent nano-filler material to improve the physical and chemical properties of materials [32,33]. Recently, Liu interestingly found that polydopamine coated CNC (PDA@CNC) as an active ingredient can enhance the adhesive force of CNC with another material while maintaining high mechanical stiffness [34]. In addition, the inherent hydrophilic properties of CNC make it suitable to be used as filling material to enhance water adsorption of composite. Therefore, we expect to realize a highly sensitive and stable QCM humidity sensor based on PDA@CNC/GO nanocomposite.

In this study, we demonstrated a high performance QCM humidity sensor based on PDA@CNC/GO composite. PDA@CNC was chemically synthesized and used as filling material in GO films to form PDA@CNC/GO nanocomposite. PDA@CNC/GO composite, acting as the sensing film, was deposited on the electrode of QCM. Humidity sensing properties of PDA@CNC/GO-coated QCM sensor, including response sensitivity, humidity hysteresis, dynamic property and stability, were systematically investigated. Moreover, the sensitivity and frequency stability of the QCM sensors based on PDA@CNC/GO, CNC/GO and GO materials were compared. Finally, the sensitive mechanism of PDA@CNC/GO-coated QCM sensor was analyzed.

## 2. Materials and Methods

### 2.1. Synthesis of Sensitive Material

**Figure 1:**
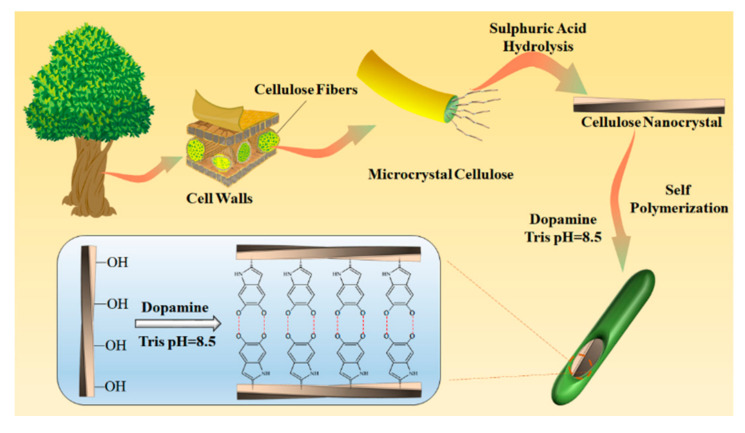
Illustration of the preparation process of PDA@CNC.

GO was synthesized by a modified Hummers method [35]. The obtained GO powder was dispersed in deionized water, and then ultrasonic treated for 2 h to obtain a homogeneous suspension. The concentration of GO suspension was 1 mg/mL. The CNC powder was supplied by ScienceK Ltd. Huzhou, China, which was prepared by sulfuric acid hydrolysis of microcrystal cellulose (MCC). The dispersion of CNC powder in deionized water was achieved by magnetic stirring for 30 min, followed by ultrasonic treatment for 2 h to form a homogeneous CNC suspension with a concentration of 3 mg/mL. The PDA@CNC was prepared by the self-polymerization of dopamine on the surface of CNC. Briefly, 3-Hydroxytyramine Hydrochloride (Adamas Reagent Co. Ltd.) (1 g) was added to CNC suspension (200 mL, 0.5 mg/mL), and the pH of the suspension was adjusted to 8.5 by adding tris(hydroxymethyl)aminomethane (Adamas Reagent Co. Ltd.). The reaction was conducted at room temperature under an air atmosphere for 24 h. The final product, PDA@CNC, was collected by centrifugation at 6000 r/min for 20 min and washed repeatedly with deionized water until the supernatant was clear. Figure 1 illustrates the preparation process of PDA@CNC. The given amount of PDA@CNC or CNC powder was added into the above GO suspension. Then, the mixture solution was ultrasonic treated for 4 h to produce homogeneous suspension, which was used as deposition solution. To learn the influence of the PDA@CNC content on the sensing properties of the resultant nanocomposite, several suspensions named PDA@CNC/GOx were prepared, where x represents the weight percentage of PDA@CNC or CNC in the GO-based nanocomposites as x wt%.

### 2.2. Sensor Fabrication

First, 10 MHz AT-cut QCMs with 8-mm crystal diameter and 5-mm electrode diameter were fabricated by Wuhan Hitrusty Electronics, Wuhan, China. The circular electrode of QCM was achieved by thermal evaporation process. A 15-nm/100-nm-thick Cr/Au layer was sequentially coated on 166-μm-thick quartz crystal plate. Prior to sensitive film deposition, the QCMs were cleaned using deionized water and ethanol, respectively. The QCM humidity sensors were fabricated by depositing an equal volume (2 μL) of suspension onto the electrode of each QCM using a micropipette. After that, these QCM sensors were placed in a drying oven and dried at 60 °C for 6 h for sufficient water evaporation. Table 1 presents the resonant parameters of the fabricated QCM humidity sensors before and after the deposition of thin film.

### 2.3. Analysis Instrument

**Figure 2:**
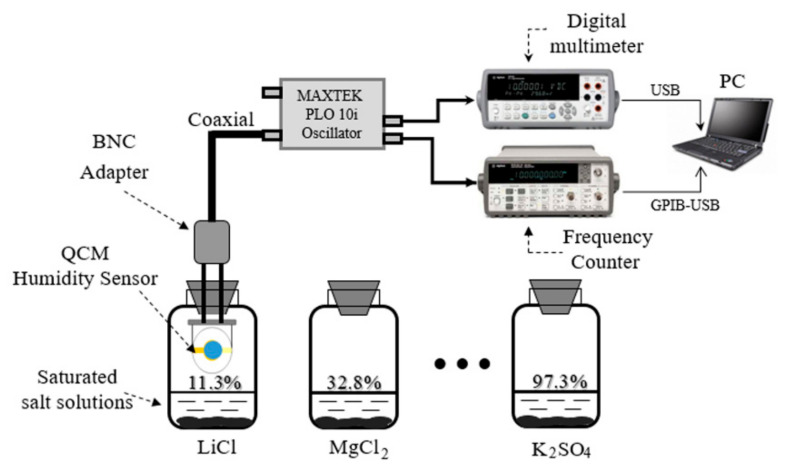
Diagram of a homemade humidity sensing measurement platform.

The homemade humidity sensing measurement platform is shown in Figure 2. It is composed of a humidity generating unit, an oscillator, a frequency counter, a digital multimeter and a PC. The required humidity points were yielded by a series of saturated salt solutions. Saturated LiCl, MgCl_2_, Mg (NO_3_)_2_, NaCl, KCl and K_2_SO_4_ solutions at 25 °C were used to generate 11.3%, 32.8%, 54.3%, 75.3%, 84.3% and 97.3% humidity levels, respectively. A commercial phase-locked loop oscillator (PLO10i, Maxtek, USA) was used to drive the QCM sensor to produce resonance. This oscillator can provide the frequency and dynamic resistance of QCM. Here, the dynamic resistance of QCM, which is inversely proportional to quality factor (*QF*)*,* is an important parameter to evaluate the stability of QCM [36]. For instance, the dynamic resistance of uncoated QCM is usually around 5–10 Ω. For the application of QCM humidity sensor, the smaller the dynamic resistance is, the better the stability is. The frequency and dynamic resistance of QCM were monitored by a frequency counter and a digital multimeter, which were connected to a PC for data acquisition and analysis. All the experiments were performed at room temperature (25 ± 1 °C). The morphology of the prepared materials was scanned by a transmission electron microscopy (TEM, Philips CM10, The Netherlands). The chemical composition of the synthesized material was characterized by Fourier transform infrared spectroscopy (FTIR, Thermo Nicolet Avatar 970 at a resolution of 8 cm^−1^ for 64 scans, WI, USA).

## 3. Results and Discussions

### 3.1. Material Characterization

**Figure 3:**
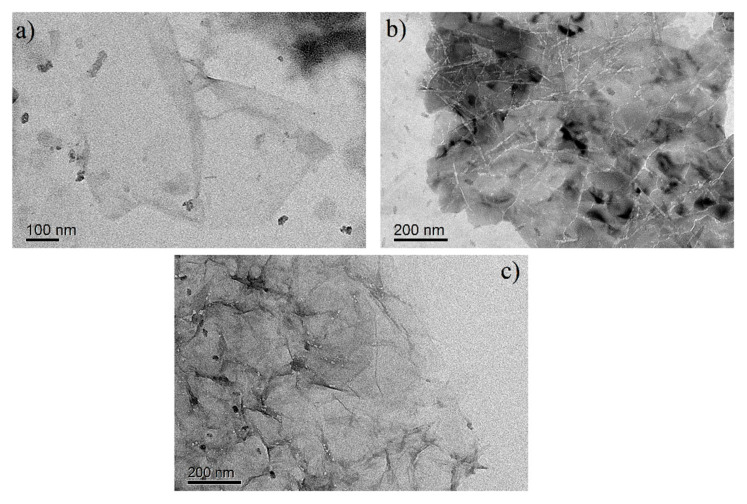
TEM images of: (**a**) GO; (**b**) CNC/GO30; and (**c**) PDA@CNC/GO30.

**Figure 4:**
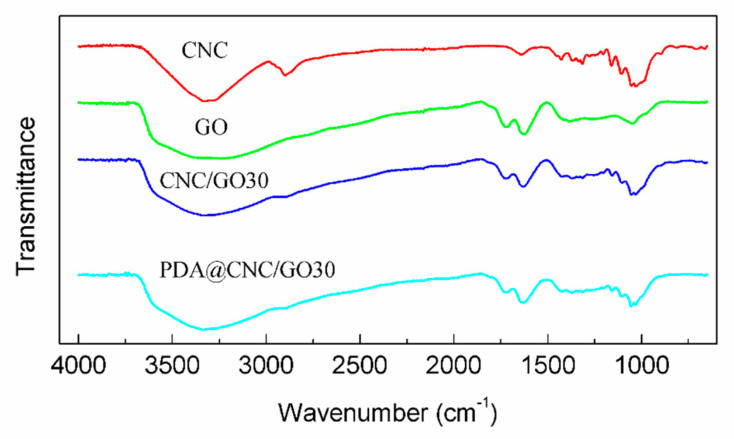
FTIR spectra of GO, CNC/GO30 and PDA@CNC/GO30 composite.

The material characteristics of the as-synthesized GO, CNC/GO30 and PDA@CNC/GO30 were analyzed by TEM, FTIR and XRD, respectively. Figure 3 shows the high resolution TEM images of GO, CNC/GO30 and PDA@CNC/GO30. It can be observed that all three samples present layered structure, which is a typical feature of two-dimensional GO materials. Figure 3b,c clearly shows the morphology of CNC/GO30 and PDA@CNC/GO30 nanocomposites. In the TEM images of the nanocomposites, it can be observed that CNC and PDA@CNC are contacted to the surface of GO sheet. Figure 4 shows the FTIR spectra of GO, CNC/GO30 and PDA@CNC/GO30. The peak appearing at 3410 cm^−1^ is attributed to the presence of –OH groups. There is a peak at 2910 cm^−1^ in the spectra of CNC and its composite resulting from the hydroxyl stretching. The two peaks at 1730 and 1640 cm^−1^ are associated with the C=O stretching and the O–H bending vibration of absorbed water. The peak appearing at 1060 cm^−1^ is due to the C−O−C pyranose ring skeletal vibrations. The FTIR analysis result confirms that the attachment of CNC and PDA@CNC to GO can be achieved through hydrogen bonding.

### 3.2. Humidity Sensing Properties of the Sensors

**Figure 5:**
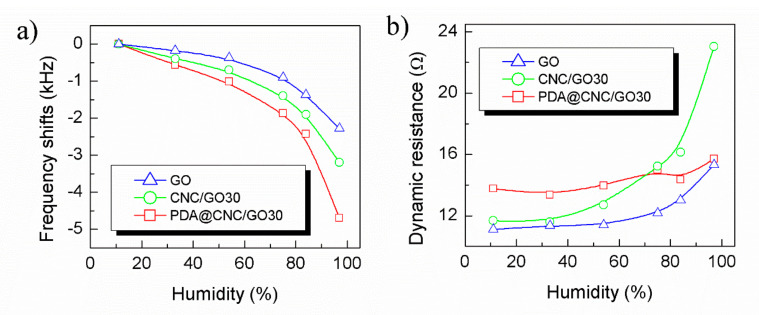
(**a**) Frequency shifts; and (**b**) dynamic resistance of GO-, CNC/GO30- and PDA@CNC/GO30-coated QCM humidity sensors as a function of RH.

The humidity sensing measurements of the QCM sensors were performed by exposing the sensors to various saturated salt solutions step by step. First, we compared the humidity sensing response and stability of the QCM humidity sensors coated with the three different sensitive films (GO, CNC/GO30 and PDA@CNC/GO30). Figure 5 shows the frequency shifts of GO-, CNC/GO30- and PDA@CNC/GO30-coated QCM humidity sensors at various humidity points. As shown in this figure, the frequency response of the three sensors exhibit a similar tendency. The frequency of the sensors decreases steadily with increasing the humidity in the humidity range of 11.3–84.3% RH. However, a steep decline in frequency is observed for all three sensors when the humidity is beyond 84.3% RH. This phenomenon is consistent with the results of most mass type humidity sensors, including micro-cantilever [7] and capacitive micro-machined ultrasonic transducer (CMUT) [8] sensors, and can be explained by water molecule cluster formation on the surface of the sensitive films according to the BET model. The observed frequency shifts shown in Figure 5 resemble the Type III sorption isotherm [15]. Regarding the three kinds of materials, the adsorption of water molecules mainly occurs at the hydrophilic functional groups (such as imino, hydroxyl and carboxyl) by hydrogen bond affinity. With the increase of adsorbed water molecules, multilayer water molecular layers are formed on the surface of sensitive materials [15]. As a result, the mass of water molecules adsorbed on the sensitive materials increases near exponentially with the increase of humidity, rather than linearly. Furthermore, it can be noted that two kinds of nanocomposite-coated (PDA@CNC/GO30 and CNC/GO30) QCM humidity sensors show an effective enhancement in frequency response compared with the pristine GO in the entire humidity range. Here, we use the ratio of the resonance frequency shifts (Δ*f*) to the humidity changes (ΔRH) to define the sensitivity of QCM humidity sensor. When humidity changes from 11.3% to 97.3% RH, the frequency shifts of PDA@CNC/GO30-, CNC/GO30- and GO-coated QCM humidity sensors were 4701, 3189 and 2279 Hz, and their corresponding sensitives are 54.66, 37.08 and 26.5 Hz/% RH, respectively. This result clearly indicates that the introduction of PDA@CNC or CNC into GO material can enhance humidity sensitivity since the filler materials offer more adsorption active sites.

**Figure 6:**
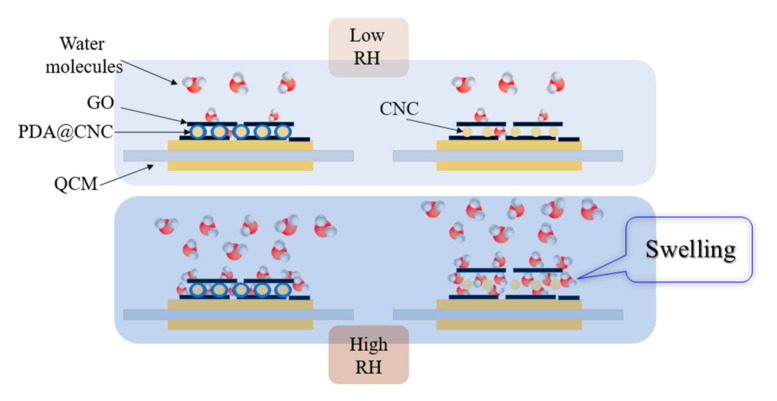
Illustration of the possible humidity sensitive mechanism of CNC/GO- and PDA@CNC/GO-coated QCM humidity sensor.

Besides the response sensitivity, dynamic resistance of the three sensors were also recorded as a function of humidity to get the evaluation of the stability [36], and the result is shown in Figure 5b. We are surprised to observe that the dynamic resistance curve of PDA@CNC/GO30-coated QCM humidity sensor exhibits a different trend compared with those of CNC/GO30- and GO-coated QCM humidity sensors. In the entire humidity range from 11.3% to 97.3% RH, the dynamic resistance of PDA@CNC/GO30-coated QCM humidity sensor exhibits very small fluctuations (rather than continuous increase) in the range of 13.77–15.7 Ω, and its change rate of dynamic resistance is only 14%. This change trend is different from most reported QCM humidity sensors. Regarding CNC/GO30- and GO-coated QCM humidity sensors, however, these dynamic resistances obviously increase with increasing humidity. Especially in the high humidity range, the dynamic resistance of CNC/GO30-coated QCM humidity sensor presents a dramatic increase. The change rates of dynamic resistance of CNC/GO30- and GO-coated QCM humidity sensors are 97.4% and 38%, which are greater than that of PDA@CNC/GO30-coated QCM humidity sensor. This valuable finding indicates that PDA@CNC is a more suitable filler material than CNC to construct high stability of QCM humidity sensor by forming nanocomposites with GO material. The observed excellent stability presented by PDA@CNC/GO30-coated QCM humidity sensor can be attributed to the inhibition of the swelling of GO-based nanocomposite by introducing PDA@CNC [34]. The adhesiveness of PDA can bind the GO lamellae tightly, thus avoiding the dramatic increase of the interlayer distance after GO adsorbs water molecules. Thus, the introduction of PDA@CNC into GO films, on the one hand, can provide more active sites for water adsorption. On the other hand, the presence of PDA@CNC into GO films can also inhibit the swelling of GO-based nanocomposite, especially after many water molecules are adsorbed on the film. Benefiting from the two advantages mentioned above, PDA@CNC/GO30-coated QCM humidity sensor possesses large sensitivity up to 54.66 Hz/% RH while holding high stability. Figure 6 illustrates the possible humidity sensitive mechanism of CNC/GO- and PDA@CNC/GO-coated QCM humidity sensor.

**Figure 7:**
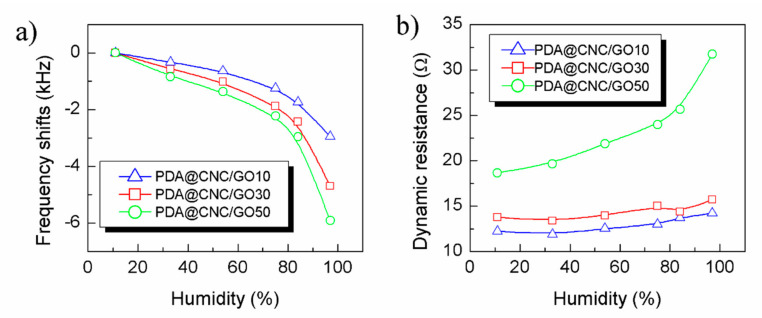
The effect of the PDA@CNC content on the performance of PDA@CNC/GO nanocomposite-coated QCM humidity sensor. (**a**) Frequency shifts and (**b**) dynamic resistance as a function of RH.

Figure 7 displays the effect of the PDA@CNC content on the performance of PDA@CNC/GO nanocomposite-coated QCM humidity sensor. The curves clearly indicate that the PDA@CNC content obviously influences frequency response sensitivity of PDA@CNC/GO nanocomposite-coated QCM humidity sensor. The frequency shifts of PDA@CNC/GO10-, PDA@CNC/GO30- and PDA@CNC/GO50-coated QCM humidity sensor are 2946, 4701 and 5905 Hz, respectively. This result further proves that the sensitivity of PDA@CNC/GO nanocomposite-coated QCM humidity sensor can be improved by increasing the PDA@CNC content. However, it should also be noted that the dynamic resistance of PDA@CNC/GO nanocomposite-coated QCM humidity sensor sharply increases when the PDA@CNC content reaches 50%, stating that the stability of such sensor reduces clearly. The reason for this phenomenon is that the viscosity of nanocomposite sharply increases when the PDA@CNC content increases to a certain value, which leads to the severe increase of the dissipation of QCM sensor. As a result, we chose PDA@CNC/GO30-coated QCM as an optimized humidity sensor under balancing the sensitivity and the stability.

**Figure 8:**
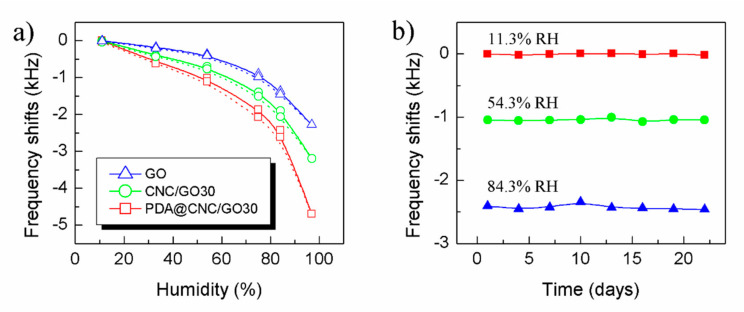
(**a**) Humidity hysteresis; and (**b**) long-term stability of PDA@CNC/GO30 nanocomposite-coated QCM humidity sensor.

Further, the other performances of PDA@CNC/GO30-coated QCM, such as humidity hysteresis, long-term stability and dynamic response, were experimentally studied. Figure 8a depicts the humidity hysteresis curves of PDA@CNC/GO30-, CNC/GO30- and GO-coated QCM humidity sensors. The solid line represents the frequency shift of the sensor from low to high RH, corresponding to the adsorption process, and the dash line indicates the frequency shifts of the sensor in the desorption process. We define the maximum humidity hysteresis rate (*HR*) in Equation (2).

(2) $$HR=\frac{\Delta f_{e}}{f_{full}}\times 100\%$$

where Δ*f_e_* is the maximum difference in frequency shifts between the adsorption and desorption process and *f_full_* is the full-scale frequency shift of the sensor. It can be calculated that the *HR* values of PDA@CNC/GO30-, CNC/GO30- and GO-coated QCM humidity sensors are 4.3%, 4.8% and 3.8%, respectively. All three kinds of GO nanocomposite-based QCM sensors exhibit relatively low humidity hysteresis, which is beneficial for the reliability of the humidity sensor in practical application. The observed small *HR* values is attributed to that the adsorption mechanism of GO-based nanocomposite is weak physical adsorption, which is conducive to water molecule adsorption or desorption of water molecules from sensitive films.

The long-term stability is also crucial for the practical application of QCM humidity sensor. We measured the frequency shifts of PDA@CNC/GO30-coated QCM humidity sensor at three given humidity points every three days for three weeks, and the result is shown in Figure 8b. It can be seen that the frequency shift curve of the sensor versus time is almost flat at low humidity point (11.3% RH) and a small fluctuation appears at high humidity (84.3% RH). In addition, the frequency fluctuations at each humidity points are calculated to be less than 6%, indicating that the prominent long-term stability of the sensor is reached.

**Figure 9:**
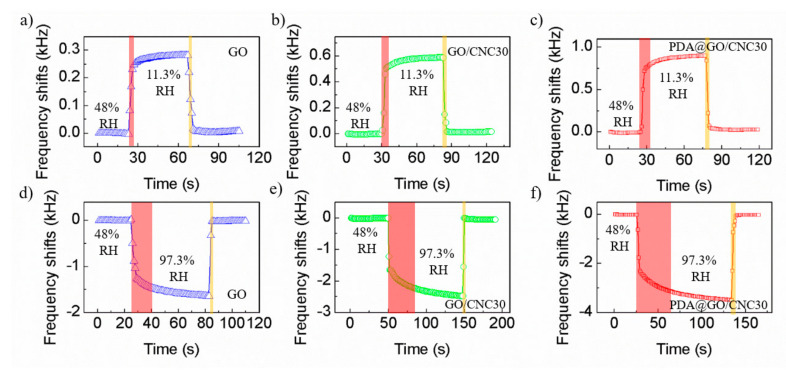
Dynamic response and recovery curves of (**a**, **d**) GO-, (**b**, **e**) CNC/GO30- and (**c**, **f**) PDA@CNC/GO30-coated QCM humidity sensors.

Figure 9 plots the dynamic response and recovery curves of the three kinds of GO nanocomposite-based QCM sensors. We measured the response and recovery of the sensor from laboratory atmosphere (~48% RH) to both low humidity point (11.3% RH) and high humidity point (97.3% RH). The response and recovery times of the sensor are defined as the time taken by the sensor to reach 90% of total output frequency shift. When the humidity pulse changes between 48% and 11.3% RH, the response/recovery times of PDA@CNC/GO30-, CNC/GO30- and GO-coated QCM humidity sensors are 11/4, 9/4 and 5/3 s, respectively. As the humidity pulse varies between 48% and 97.3% RH, the corresponding response/recovery times of the sensors are 37/5, 32/3 and 16/3 s, respectively. The response and recovery speed of these GO nanocomposite-coated QCM sensors in the low and middle humidity range is faster than that of high humidity range. The introduction of PDA@CNC or CNC into GO material inevitably increases response time of the sensor, especially when the detection humidity changes to high humidity. Although the response time of PDA@CNC/GO30-coated QCM is larger than that of GO-coated QCM, it is also superior to many reported polymer-coated QCM humidity sensors [26,29] and can be used in most humidity detection situations. Additionally, it is worth noting that the recovery speed of these GO nanocomposite-coated QCM sensors is very fast whether from low RH to laboratory atmosphere or high RH to laboratory atmosphere. This result also implies that the water molecules adsorption and desorption of GO nanocomposite can more easily achieve a dynamic equilibrium under medium humidity conditions. Table 2 summarizes the performance comparison between the PDA@CNC/GO nanocomposite-coated QCM humidity sensor and some recent state-of-the-art humidity sensors reported in the literature. It can be seen that PDA@CNC/GO nanocomposite-coated QCM humidity sensor exhibits a larger sensitivity than most reported QCM humidity sensors. More importantly, our sensor overcomes the disadvantage that QCM humidity sensors usually have of poor stability at high humidity.

## 4. Conclusions

The present work demonstrates a high performance QCM humidity sensor using PDA@CNC/GO nanocomposite as sensitive material. Series of experiments were carried out to examine humidity sensing performance of the proposed sensor, including sensitivity, stability, humidity hysteresis, dynamic response and long-term stability. The sensitivity of PDA@CNC/GO30-coated QCM humidity sensor was about two times larger than that of GO-coated QCM sensor. More interestingly, we observed that the change rate of dynamic resistance of PDA@CNC/GO30-coated QCM humidity sensor is only 14%, which was much smaller than that of CNC/GO30- and GO-coated QCM sensors. The results evidently show that the introduction of PDA@CNC into GO films can significantly improve the sensor’s sensitivity while maintaining high stability in the entire humidity range. The explanation of the above experimental results is that the introduction of PDA@CNC into GO films not only provided more hydrophilic groups for water adsorption but also inhibited the swelling of GO-based nanocomposite after water adsorption. Furthermore, the effect of the PDA@CNC content on the performance of GO nanocomposite-coated QCM humidity was also considered. In addition, PDA@CNC/GO30-coated QCM humidity sensor possessed an acceptable humidity hysteresis (4.3% RH), fast response and recovery (37/5 s at 48–97.3% RH) and prominent long-term stability. Our work suggests that PDA@CNC/GO nanocomposite is a promising candidate material for realizing high sensitivity and stability QCM humidity sensors in the entire humidity detection range.

## Figures and Tables

**Table 1:** Resonant parameters of QCM sensor before and after the deposition of thin film.

Mark Number	Sensing Material	Before the Deposition of Thin Film		After the Deposition of Thin Film	
		Frequency (Hz)	Dynamic Resistance (Ω)	Frequency (Hz)	Dynamic Resistance (Ω)
GO-coated QCM	GO	9,984,827	11.0945	9,978,865	11.1042
CNC/GO30-coated QCM	CNC/GO30	9,984,811	10.349	9,975,052	11.6756
PDA@CNC/GO30-coated QCM	PDA@CNC/GO30	9,984,821	11.0945	9,972,163	13.7241

**Table 2:** Performance comparison between the proposed sensor in this work and other recent state-of-the-art humidity sensors.

Sensing Material	Sensor Type	Sensitivity (ppm/% RH)/Range	Response and Recovery Times (s)	Dynamic Resistance (Ω)	Hysteresis (% RH)	Ref
GO	CMUT	241.67/22.5–43.2% RH	10/4 s	Not given	Not given	[8]
MWCNTs-CS	QCM	4.67/11–95% RH	75/34 s	51.92@11% RH 151.32@95% RH	1.1	[20]
GO-PEI	QCM	2.73/11–97% RH	53/18 s	Not given	0.54	[26]
GO/SnO2/PANI	QCM	3.64/0–97% RH	7/2 s	Not given	Not given	[29]
MWCNTs-GO	QCM	0.98/10–95% RH	12/6 s	42.8164@10% RH 96.2581@95% RH	Not given	[37]
TiO_2_ nano-particles	QCM	0.75/0–20% RH	2/4 min	Not given	<2	[38]
γ-Al_2_O_3_	SAW	0.28/3–20% RH	1/3 s	Not given	0.3	[39]
Chitosan@ZONRs	Cantilever	16.9/30–70% RH	1 s/Not given	Not given	2.1	[40]
PDA@CNC/GO	QCM	5.466/11.3–97.3% RH	11/4 s (from 48% to 11.3% RH) 37/5 s (from 48% to 97.3% RH)	13.7724@11.3% RH 15.7015@97.3% RH	4.3	This work
